# *GJB2 *mutation spectrum in 2063 Chinese patients with nonsyndromic hearing impairment

**DOI:** 10.1186/1479-5876-7-26

**Published:** 2009-04-14

**Authors:** Pu Dai, Fei Yu, Bing Han, Xuezhong Liu, Guojian Wang, Qi Li, Yongyi Yuan, Xin Liu, Deliang Huang, Dongyang Kang, Xin Zhang, Huijun Yuan, Kun Yao, Jinsheng Hao, Jia He, Yong He, Youqin Wang, Qing Ye, Youjun Yu, Hongyan Lin, Lijia Liu, Wei Deng, Xiuhui Zhu, Yiwen You, Jinghong Cui, Nongsheng Hou, Xuehai Xu, Jin Zhang, Liang Tang, Rendong Song, Yongjun Lin, Shuanzhu Sun, Ruining Zhang, Hao Wu, Yuebing Ma, Shanxiang Zhu, Bai-lin Wu, Dongyi Han, Lee-Jun C Wong

**Affiliations:** 1 Department of Otolaryngology and Genetic Testing Center for Deafness, Chinese PLA General Hospital, Beijing 100853, PR China; 2 Department of Molecular and Human Genetics, Baylor College of Medicine, Houston, Texas, USA; 3 Department of Otolaryngology, University of Miami, Miami, FL 33136, USA; 4 Department of Otolaryngology, Fuyang People's Hospital, Fuyang 157011, Anhui, PR China; 5 Department of Otolaryngology, Beijing Children's Hospital, Beijing 100045, PR China; 6 Department of Health Statistics, Second Military Medical University, Shanghai, PR China; 7 Department of Otolaryngology, Fuzhou Second People's Hospital, Fuzhou 528000, Fujian, PR China; 8 Center of Hearing Rehabilitation, Guizhou People's Hospital, Guiyang 550002, Guizhou, PR China; 9 Department of Otolaryngology, Foshan First People's Hospital, Foshan 528041, Guangdong, PR China; 10 Department of Otolaryngology, Anyang Stomatology Hospital, Anyang 455000, Henan, PR China; 11 Department of Otolaryngology, Mudanjiang First People's Hospital, Mudanjiang 157011, Heilongjiang, PR China; 12 Department of Otolaryngology, PLA 161st Hospital, Wuhan 430010, Hubei, PR China; 13 Department of Otolaryngology, Chifeng Second People's Hospital, Chifeng 024000, Inner Mongolia, PR China; 14 Department of Otolaryngology, Affiliated Hospital of Nantong University, Nantong 226001, Jiangsu, PR China; 15 Department of Otolaryngology, Affiliated Hospital of Beihua University, Jilin 132011, Jilin, PR China; 16 Department of Otolaryngology Head&neck Surgery, General Hospital of Lanzhou Area Command, Lanzhou 730050, Gansu, PR China; 17 Department of Otolaryngology, Urumchi People's Hospital, Urumchi 830001, Xinjiang, PR China; 18 Department of Otolaryngology, Zhuozhou Second Central Hospital, Zhuozhou 072750, Hebei, PR China; 19 Department of Otolaryngology, Datong Third People's Hospital, Datong 037008, Shanxi, PR China; 20 Department of Otolaryngology, Yuncheng Central Hospital, Yuncheng 044000, Shanxi, PR China; 21 Department of Otolaryngology Head & Neck Surgery, Affiliated Xinhua Hospital of Shanghai Jiao Tong University, Shanghai, 200092, PR China; 22 Department of Otolaryngology, General Hopital of Tibet Area Command, Lhasa 850000, Tibet, PR China; 23 Institute of Geriatrics, Chinese PLA General Hospital, Beijing 100853, PR China; 24 Division of Genetics and Metabolism, Children's Hospital Boston, Harvard Medical School, Boston, Massachusetts, USA

## Abstract

**Background:**

Mutations in *GJB2 *are the most common molecular defects responsible for autosomal recessive nonsyndromic hearing impairment (NSHI). The mutation spectra of this gene vary among different ethnic groups.

**Methods:**

In order to understand the spectrum and frequency of *GJB2 *mutations in the Chinese population, the coding region of the *GJB2 *gene from 2063 unrelated patients with NSHI was PCR amplified and sequenced.

**Results:**

A total of 23 pathogenic mutations were identified. Among them, five (p.W3X, c.99delT, c.155_c.158delTCTG, c.512_c.513insAACG, and p.Y152X) are novel. Three hundred and seven patients carry two confirmed pathogenic mutations, including 178 homozygotes and 129 compound heterozygotes. One hundred twenty five patients carry only one mutant allele. Thus, *GJB2 *mutations account for 17.9% of the mutant alleles in 2063 NSHI patients. Overall, 92.6% (684/739) of the pathogenic mutations are frame-shift truncation or nonsense mutations. The four prevalent mutations; c.235delC, c.299_c.300delAT, c.176_c.191del16, and c.35delG, account for 88.0% of all mutantalleles identified. The frequency of *GJB2 *mutations (alleles) varies from 4% to 30.4% among different regions of China. It also varies among different sub-ethnic groups.

**Conclusion:**

In some regions of China, testing of the three most common mutations can identify at least one *GJB2 *mutant allele in all patients. In other regions such as Tibet, the three most common mutations account for only 16% the *GJB2 *mutant alleles. Thus, in this region, sequencing of *GJB2 *would be recommended. In addition, the etiology of more than 80% of the mutant alleles for NSHI in China remains to be identified. Analysis of other NSHI related genes will be necessary.

## Introduction

Hearing impairment is the most common neurosensory disorder in humans. The reported incidence varies from 1 in 300 to 1 in 1000 children [1-4]. Approximately half of cases have a genetic etiology, including syndromic and non-syndromic forms, with extraordinary genetic heterogeneity. Non-syndromic deafness accounts for 60–70% of inherited hearing impairment. It involves more than 100 different genes with autosomal dominant (DFNA), autosomal recessive (DFNB), X-linked (DFN), and maternal inheritance [5], with autosomal recessive being the most common. For many populations, the most common cause for non-syndromic autosomal recessive hearing loss is mutated Connexin 26, a gap junction protein encoded by the *GJB2 *gene (MIM 121011) [6-13]. There are a few specific mutations in *GJB2 *gene that are associated with the autosomal dominant syndromic forms of deafness, and typically present with skin abnormalities including keratitis-ichthyosis [14-16].

Connexins are transmembrane proteins. Six monomers of connexin proteins associate to form a transmembrane hexameric gap junction hemi-channel called a connexon. Connexons embedded in the surfaces of adjacent cells associate to form an intercellular channel [17,18]. In the inner ear, connexin 26 can be in association with other connexins to form heteromeric connexons. Gap junction channels can be homotypic or heterotypic. Connexin 26 gap junction channels recycle potassium ions as part of a mechanism of auditory signal transduction in inner ear [19].

Mutations in three connexin (Cx) genes, *GJB2 *(Cx26), *GJB6 *(Cx30), and *GJB3 *(Cx31), have been identified and are known to cause hearing impairment [18,19]. Sequence analysis of the *GJB2 *gene in subjects with autosomal recessive hearing impairment revealed that a high number of patients carried only one mutant allele. Some of these families showed clear evidence of linkage to the DFNB1 locus, which contains two genes, *GJB2 *and *GJB6 *[6,20]. Further analysis demonstrated that some *GJB2 *heterozygotes also carried a truncating deletion of the *GJB6 *gene, encoding connexin 30, in *trans *[21,22].

To date, more than 150 mutations, polymorphisms, and unclassified variants have been described in the *GJB2 *gene . The mutation spectrum and prevalence of mutations vary significantly among different ethnic groups. Three mutations, c.35delG, c.167delT, and c.235delC, are found to be the most frequent mutations in Caucasian, Ashkenazi Jewish, and Asian populations, respectively [6,7,9-13,20,23-26].

**Figure 1:**
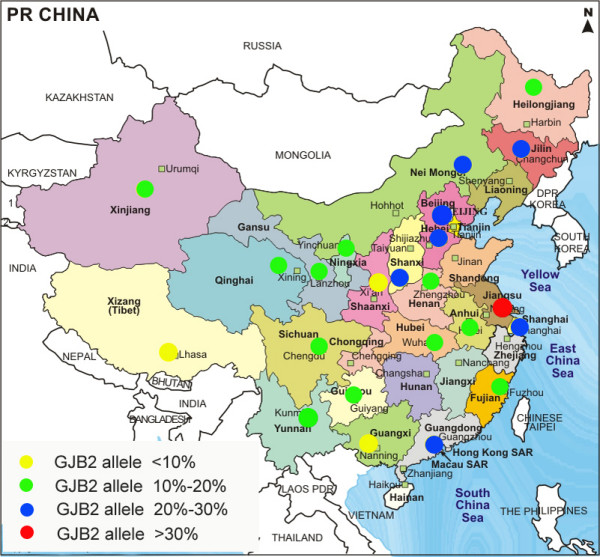
**Geographic distribution and the proportion of patients carrying at least one *GJB2 *mutant allele in each region studied**.

In China, it is estimated that 30,000 babies are born with congenital hearing impairment every year [27]. The mutation spectrum of the *GJB2 *gene in Chinese patients with nonsyndromic hearing impairment (NSHI) has not been analyzed. Our recent study by screening for just the most common mutation, c.235delC, in 3004 Chinese NSHI patients revealed that 488 (16.3%) patients carried at least one c.235delC mutant allele, with 233 (7.8%) homozygotes and 255 (8.5%) heterozygotes [28], though the frequencies of homozygote and heterozygote of c.235delC varied from 0% to 14.7% and from 1.7% to 16.1% respectively in the populations examined in this study. Among different Chinese sub-ethnic groups the c.235delC allele frequency was the lowest (0.8%) in the Tibetan and the highest (31.0%) in Maan. These results highlight the need to sequence the entire *GJB2 *gene in order to more accurately establish the actual mutation frequency and mutation spectrum of *GJB2 *gene within various Chinese sub-populations. Our preliminary results reveal that other *GJB2 *mutations account for an additional 7.1% of NSHI patients from Qinghai, where only 7.1% patients carried at least one c.235delC mutation. Nevertheless, sequencing analysis of the entire coding region of the *GJB2 *gene in patients from Guangxi where the frequency of the c.235delC mutation is 3.4% reveals only one other mutation in 87 deaf patients. These results have two important implications: that the *GJB2 *gene needs to be sequenced in its entirety; and that mutations in genes responsible for NSHI other than *GJB2 *should be searched in patients who do not harbor two mutant alleles in the *GJB2 *gene. In this study, we report the results of sequencing the *GJB2 *gene in 2063 patients with NSHI from 23 different regions of China (Figure 1).

## Materials and methods

### Patients and DNA samples

A total of 2063 unrelated NSHI students from 23 different regions of China were included in this sequencing study. The selection of samples was random regardless of the c.235delC genotype. The patients consisted of 1179 males and 884 females ranging in age from 2 to 30 years with an average age of 13.7 ± 4.5. The majority of patients were Han Chinese (1640), followed by Tibetan (122), minorities in the Southwest region (119), Hui (79), minorities in Xinjiang (62), Mongolian (21), Maan (18) and Korean (2). Ethnic subgroup designations were based on permanent residency documentation.

This study was performed according to a protocol approved by the ethics committee of the Chinese PLA General Hospital. The subjects in this study were from deaf schools of each region and were recently described [28]. Only the unrelated patients with nonsyndromic hearing loss were included in this study. Parents were not included in this study. All patients showed moderate to profound bilateral sensorineural hearing impairment on audiograms and no pathient with mild hearing impairment was found in this cohort. In addition to the 2063 patients, 301 Han control individuals with normal hearing (either evaluated by pure tone audiometry or by self-assessment) from Beijing Capital (Northern) and Jiangsu Province (Eastern), two densely populated regions consisting of 98% Han Chinese, were also analyzed. DNA was extracted from peripheral blood leukocytes using a commercially available DNA extraction kit (Watson Biotechnologies Inc, Shanghai, China).

### Sequence analysis

The coding exon (Exon2) and flanking intronic regions of *GJB2 *gene were PCR amplified with forward primer 5'TTGGTGTTTGCTCAGGAAGA 3' and reverse primer 5'GGCCTACAGGGGTTTCAAAT 3'. Among this study cohort, 851 patients from central China were also analyzed for mutations in Exon1 and flanking introns by PCR/sequencing. The PCR primers used are forward primer: 5'CTCATGGGGGCTCAAAGGAACTAGGAGATCGG3' and reverse primer 5'GGGGCTGGACCAACACACGTCCTTGGG3'. The PCR products were purified on Qia-quick spin columns (Qiagen, Valencia, CA) and sequenced using the BigDye Terminator Cycle Sequencing kit (version v3.1) and ABI 3130 automated DNA sequencers (Applied Biosystems, Foster City, CA, USA,) with Sequence Analysis Software (Sequencing Analysis version 3.7). DNA sequence variations were identified by comparison of subject DNA sequence to *GJB2 *reference sequences, Genebank Accession Number AY280971. Numbering of *GJB2 *begins with the nucleotide A of the ATG start codon in Exon2 as cDNA position number 1. The sequences were analyzed using Genetool Lite software and the *GJB2 *Genebank sequence. The presence of 309 kb deletion of *GJB6 *was analyzed by PCR method [21,22]. A positive control of this deletion provided by Balin Wu (Department of Laboratory Medicine, Children's Hospital and Harvard Medical School, USA.) was used for the detection of deletion in *GJB*6 gene.

### Statistical analysis

The statistical analysis was performed using SAS 9.1.3 software (SAS, Cary, North Carolina, USA).

## Results

### Mutations in *GJB2 *gene

Sequencing of the coding region of the *GJB2 *gene revealed that at least 104 different genotypes were found in the 2063 patients (Table 1). Among them, 64 different genotypes harboring pathogenic mutations were found in 432 patients (Table 1). Three hundred and seven patients had two confirmed pathogenic mutations, including 178 homozygotes and 129 compound heterozygotes. One hundred twenty five patients carried one heterozygous pathogenic mutation without an identified second mutant allele. Thus, *GJB2 *mutant alleles account for 17.9% (739/4126) of the total alleles in 2063 NSHI patients. The most common genotype was homozygous c.235delC, followed by compound heterozygosity for c.235delC/c.299_300delAT, which accounted for 8.0% (164/2063) and 3.2% (66/2063) of NSHI patients respectively. The most common mutation c.235delC was in compound heterozygosity with 14 other different pathogenic mutations in 113 patients, and was present as a single heterozygous mutant allele in 68 patients. In addition, there were 23 different genotypes in patients carrying one allele of unclassified variants (Table 1). Twenty-three alterations were found, five (p.W3X, c.99delT, c.155_c.158delTCTG, p.Y152X, and c.512_c.513insAACG) of them were novel and pathogenic, and twelve (p,G21R, p,I30F, p.F31L, p.V37I, p.V63L, p.T123N, p.V153A, p.D159N, p.F191L, p.M195V, p.V198M, and p.I215N) are unclassified variants (Table 1 and Supplemental Table 1). The distribution of various genotypes in 23 regions (Figure 1) is detailed in Table 2 and Supplemental Table 2. The frequencies of the three most common *GJB2 *mutations in the 23 regions studied are listed in Table 2. The allele frequency of all mutations in the *GJB2 *gene in NSHI patients varied from 4.0% in Guangxi to 30.4% in Jiangsu (Table 2). Regions which appeared to have a higher frequency of the c.235delC mutation (Jiangsu, Inner Mongolia, Beijing, Hebei, Shanghai) also had a relatively high frequency of other *GJB2 *mutations (eg, the frequency of the c.235delC mutation in Jiangsu was as high as 20.6% and the frequencies of other mutations were also as high as 9.8%). Similarly, regions such as Shaanxi and Guangxi where the frequency of the c.235delC mutation is low (5.8 and 3.4% respectively), also had lower frequencies of other mutations (1.9 and 0.6% respectively). Patients from Tibet, Yunnan, Xinjiang, Heilongjiang, and Ningxia appear to have the most diverse mutation spectrum because uncommon mutations (except c.235delC, c.299_c.300delAT and c.176_c.191del16) comprise 84.2, 30.8, 26.1, 21.4, and 20.4%, respectively of overall *GJB2 *mutations in those regions.

**Table 1:** *GJB2 *genotypes of 2063 Chinese NSHI patients

Allele 1				Allele 2				
nucleotide change	consequence or amino acid change	category	domain	nucleotide change	consequence or amino acid change	Category	domain	Number of patients^d^
								
homozygous								
c.35delG	frame-shift	pathogenic	NT	c.35delG	frame-shift	Pathogenic		2
c.176_c.191del16	fram shift	pathogenic	EC1	c.176_c.191del16	frame-shift	Pathogenic	EC1	2
c.235delC	frame-shift	pathogenic	TM2	c.235delC	frame-shift	Pathogenic	TM2	164
c.299_c.300delAT	frame-shift	pathogenic	CL	c.299_c.300delAT	frame-shift	pathogenic	CL	8
c.512_c.513insAACG	frame-shift	pathogenic	EC2	c.512_c.513insAACG	frame-shift	pathogenic	EC2	1
c.605_c.606ins46	frame-shift	pathogenic	TM4	c.605_c.606ins46	frame-shift	pathogenic	TM4	1

compound heterozygous								
c.9G>A, c.79G>A	p.W3X, p.V27I	pathogenic, polymophism	NT, TM1	c.427C>T	p.R143W	pathogenic	TM3	1
c.35delG	frame-shift	pathogenic	NT	c.299_c.300delAT	frame-shift	pathogenic	CL	1
c.35delG	frame-shift	pathogenic	NT	c.313_c.326del14	frame-shift	pathogenic	CL	1
c.176_c.191del16	frame-shift	pathogenic	EC1	c.9G>A, c.79G>A	p.W3X, p.V27I	pathogenic, polymophism	NT+TM1	2
c.176_c.191del16	frame-shift	pathogenic	EC1	c.299_c.300delAT	frame-shift	pathogenic	CL	4
c.176_c.191del16	frame-shift	pathogenic	EC1	c.388_c.397del10	frame-shift	pathogenic		1
c.235delC	frame-shift	pathogenic	TM2	c.9G>A, c.79G>A	p.W3X, p.V27I	pathogenic, polymophism	NT+TM1	2
c.235delC	frame-shift	pathogenic	TM2	c.35delG	frame-shift	pathogenic	NT	1
c.235delC	frame-shift	pathogenic	TM2	c.35insG	frame-shift	pathogenic	NT	2
c.235delC	frame-shift	pathogenic	TM2	c.94C>T	p.R32C	pathogenic	TM1	1
c.235delC	frame-shift	pathogenic	TM2	c.99delT	frame-shift	pathogenic	TM1	1
c.235delC	frame-shift	pathogenic	TM2	c.139G>T	p.E47X	pathogenic	EC1	3
c.235delC	frame-shift	pathogenic	TM2	c.155_c.158delTCTG	frame-shift	pathogenic	EC1	2
c.235delC	frame-shift	pathogenic	TM2	c.176_191del16	frame-shift	pathogenic	EC1	18
c.235delC	frame-shift	pathogenic	TM2	c.257C>G	p.T86R	pathogenic	TM2	6
c.235delC	frame-shift	pathogenic	TM2	c.299_c.300delAT	frame-shift	pathogenic	CL	65
c.235delC	frame-shift	pathogenic	TM2	c.299_c.300delAT, c.79G>A	frame-shift, p.V27I	frame-shift, polymorphism	CL+TM1	1
c.235delC	frame-shift	pathogenic	TM2	c.313_c.326del14	frame-shift	pathogenic	CL	1
c.235delC	frame-shift	pathogenic	TM2	c.427C>T	p.R143W	pathogenic	TM3	3
c.235delC	frame-shift	pathogenic	TM2	c.512_c.513insAACG	frame-shift	pathogenic	EC2	6
c.235delC	frame-shift	pathogenic	TM2	c.605_c.606ins46	frame-shift	pathogenic	TM4	1
c.299_c.300delAT	frame-shift	pathogenic	CL	c.139G>A	p.E47K	pathogenic	EC1	1
c.299_c.300delAT	frame-shift	pathogenic	CL	c.257C>G	p.T86R	pathogenic	TM2	1
c.299_c.300delAT	frame-shift	pathogenic	CL	c.512_c.513insAACG	frame-shift	pathogenic	EC2	3
c.456C>A	p.Y152X	pathogenic	TM3, CL	c.380G>A, c.79G>A, c.341A>G	p.R127H, p.V27I, E114G	pathogenic, polymophism	TM1+CL	1

heterozygous (one mutant allele only)								
c.11G>A	p.G4D	pathogenic	NT	c.109G>A	p.V37I	see note	TM1	1
c.11G>A	p.G4D	pathogenic	NT	Nv				2
c.35delG	frame-shift	pathogenic	NT	c.79G>A, c.341A>G	p.V27I p,E114G	polymorphism	TM1+CL	1
c.35delG	frame-shift	pathogenic	NT	Nv				4
c.155_c.158delTCTG	frame-shift	pathogenic	EC1	c.341A>G, c.644T>A	p.E114G, p.I215N	polymorphism, unclassified	CL+CT	1
c.176_c.191del16	frame-shift	pathogenic	EC1	Nv				2
c.235delC	frame-shift	pathogenic	TM2	c.109G>A	p.V37I	see note	TM1	11
c.235delC	frame-shift	pathogenic	TM2	c.79G>A	p.V27I	polymorphism	TM1	6
c.235delC	frame-shift	pathogenic	TM2	c.79G>A, c.341A>G	p.V27I, p.E114G	polymorphism	TM1+CL	5
c.235delC	frame-shift	pathogenic	TM2	c.341A>G	p.E114G	polymorphism	CL	2
c.235delC	frame-shift	pathogenic	TM2	c.558G>A	p.T186T	polymorphism	EC2	1
c.235delC	frame-shift	pathogenic	TM2	Nv				43
c.253T>C	p.S85P	pathogenic	TM2	Nv				1
c.299_c.300delAT	frame-shift	pathogenic	CL	c.109G>A	p.V37I	see note	TM1	1
c.299_c.300delAT	frame-shift	pathogenic	CL	c.79G>A, c.341A>G	p.V27I, p.E114G	polymorphism	TM1+CL	1
c.299_c.300delAT	frame-shift	pathogenic	CL	Nv				4
c.380G>A, c.341A>G	p.R127H, p.E114G	pathogenic, polymophism	CL+CL	c.109G>A	p.V37I	see note	TM1	1
c.380G>A	p.R127H	pathogenic	CL	c.109G>A	p.V37I	see note	TM1	1
c.380G>A, c.109G>A	p.R127H, p.V37I	pathogenic, polymophism	TM1+CL	c.79G>A	p.V27I	polymorphism	TM1	1
c.380G>A, c.79G>A	p.R127H, p.V27I	pathogenic, polymophism	TM1+CL	c.79G>A, c.341A>G	p.V27I, p.E114G	polymorphism	TM1+CL	1
c.380G>A	p.R127H	pathogenic	CL	c.79G>A, c.341A>G	p.V27I, p.E114G	polymorphism	TM1+CL	9
c.380G>A, c.147C>T	p.R127H, p.A49A	pathogenic, polymophism	EC1+CL	c.79G>A	p.V27I	polymorphism	TM1	1
c.380G>A, c.608T>C	p.R127H, p.I203T	pathogenic, polymophism	CL+TM4	c.79G>A, c.341A>G	p.V27I, p.E114G	polymorphism	TM1+CL	1
c.380G>A, c.608T>C	p.R127H, p.I203T	pathogenic, polymophism	CL+TM4	c.79G>A	p.V27I	polymorphism	TM1	1
c.380G>A	p.R127H	pathogenic	CL	c.79G>A	p.V27I	polymorphism	TM1	4
c.380G>A	p.R127H	pathogenic	CL	c.457G>A	p.V153I	polymorphism	TM3	1
c.380G>A	p.R127H	pathogenic	CL	Nv				10
c.416G>A	p.S139N	pathogenic	CL	c.79G>A, c.341A>G	p.V27I, p.E114G	polymorphism	TM1+CL	1
c.416G>A	p.S139N	pathogenic	CL	Nv				1
c.424_c.426del3	p.del142F	pathogenic	TM3	c.79G>A, c.341A>G, c.109G>A	p.V27I, p.E114G, p.V37I	polymorphisms, see note	TM1+CL	3
c.424_c.426del3	p.del142F	pathogenic	TM3	c.79G>A, c.109G>A	p.V27I, p.V37I	polymorphisms, see note	TM1	1
c.512_c.513insAACG	frame-shift	pathogenic	EC2	c.79G>A, c.368C>A	p.V27I, p.T123N	polymorphism, unclassified	TM1+CL	1
c.512_c.513insAACG	frame-shift	pathogenic	EC2	Nv				1

unclassified variant								
c.61G>C, c.79G>A	p.G21R, p.V27I	unclassified, polymorphism	NT+TM1	c.79G>A, c.341A>G	p.V27I, p.E114G	polymorphism	TM1+CL	1
c.88A>T	p.I30F	unclassified	TM1	Nv				1
c.93T>G	p.F31L	unclassified	TM1	c.79G>A, c.341A>G	p.V27I, p.E114G	polymorphism	TM1+CL	1
c.187G>T	p.V63L	unclassified	EC1	Nv				2
c.368C>A, c.79G>A	p.T123N, p.V27I	unclassified, polymorphism	CL+TM1	c.79G>A, c.341A>G	p.V27I, p.E114G	polymorphism	TM1+CL	1
c.368C>A, c.79G>A	p.T123N, p.V27I	unclassified, polymorphism	CL+TM1	c.79G>A	p.V27I	polymorphism	TM1	3
c.368C>A	p.T123N	unclassified	CL	c.79G>A	p.V27I	polymorphism	TM1	7
c.368C>A, c.608T>C	p.T123N, p.I203T	unclassified, polymorphism	CL+TM4	c.79G>A	p.V27I	polymorphism	TM1	1
c.458T>C	p.V153A	unclassified	EC2	c.608T>C	p.I203T	polymorphism	TM4	1
c.571T>C, c.592G>A	p.F191L, p.V198M	unclassified	TM4+TM4	c.79G>A	p.V27I	polymorphism	TM1	1
c.583A>G	p.M195V	unclassified	TM4	Nv				1
c.583A>G	p.M195V	unclassified	TM4	c.79G>A, c.341A>G	p.V27I, p.E114G	polymorphism	TM1+CL	1
c.592G>A, c.79G>A, c.341A>G	p.V198M, p.V27I, p.E114G	unclassified, polymorphism	TM4+TM1+CL	c.79G>A, c.341A>G	p.V27I, p.E114G	polymorphism	TM1+CL	1
c.592G>A, c.79G>A	p.V198M, p.V27I	unclassified, polymorphism	TM4+TM1	c.79G>A, c.341A>G	p.V27I, p.E114G	polymorphism	TM1+CL	1
c.592G>A	p.V198M	unclassified	TM4	c.79G>A, c.341A>G	p.V27I, p.E114G	polymorphism	TM1+CL	2
c.475G>A	p.D159N	unclassified	EC2	Nv			TM1+CL	1
c644T>A, c.79G>A, c.341A>G	p.I215N, p.V27I, p.E114G	unclassified, polymorphism	CT+TM1+CL	c.79G>A, c.341A>G	p.V27I, p.E114G	polymorphism	TM1+CL	1
c.644T>A	p.I215N	unclassified	CT	c.608T>C	p.I203T	polymorphism	TM4	1
c.109G>A	p.V37I	see note	TM1	c.109G>A	p.V37I	see note	TM1	23
c.109G>A	p.V37I	see note	TM1	c.79G>A, c.341A>G	p.V27I, p.E114G	polymorphism	TM1+CL	29
c.109G>A	p.V37I	see note	TM1	c.79G>A	p.V27I	polymorphism	TM1	10
c.109G>A	p.V37I	see note	TM1	c.608T>C	p.I203T	polymorphism	TM4	3
c.109G>A	p.V37I	see note	TM1	Nv				91

polymorphism								
c.79G>A, c.341A>G	p.V27I, p.E114G	polymorphism	TM1+CL	c.79G>A, c.341A>G	p.V27I, p.E114G	polymorphism	TM1+CL	90
c.79G>A	p.V27I	polymorphism	TM1	c.79G>A	p.V27I	polymorphism	TM1	18
c.79G>A, c.341A>G	p.V27I, p.E114G	polymorphism	TM1+CL	c.79G>A	p.V27I	polymorphism	TM1	42
c.79G>A, c.341A>G	p.V27I, p.E114G	polymorphism	TM1+CL	c.341A>G	p.E114G	polymorphism		2
c.79G>A, c.341A>G	p.V27I, p.E114G	polymorphism	TM1+CL	c.457G>A	p.V153I	polymorphism	TM3	1
c.79G>A, c.341A>G	p.V27I, p.E114G	polymorphism	TM1+CL	c.608T>C	p.I203T	polymorphism	TM4	12
c.79G>A, c.341A>G	p.V27I, p.E114G	polymorphism	TM1+CL	Nv				387
c.79G>A	p.V27I	polymorphism	TM1	c.608T>C	p.I203T	polymorphism	TM4	5
c.79G>A, c.608T>C	p.V27I	polymorphism	TM1+TM4	c.608T>C	p.I203T	polymorphism	TM4	1
c.79G>A	p.V27I	polymorphism	TM1	Nv				202
c.147C>T	p.A49A	polymorphism	EC1	Nv				1
c.181A>G	p.K61K	polymorphism	EC1	Nv				1
c.341A>G	p.E114G	polymorphism	CL	Nv				14
c.438C>T	p.F146F	polymorphism	TM3	Nv				2
c.608T>C	p.I203T	polymorphism	TM4	c.608T>C	p.I203T	polymorphism	TM4	3
c.608T>C	p.I203T	polymorphism	TM4	Nv				28

nv				Nv				638

total								2063

nv: no variant

Note: p.V37I is controversy variant, see the discussion.

**Table 2:** Prevalence of *GJB2 *mutations in different areas of China

		Number of NSHI			c.235delC allele			c.299_c.300delAT allele			c.176_c.191del16 allele			Uncommon mutant allele			total number of mutant alleles(%)
	total	with two mutation	1 allele with one mutaion	number with 1 mutant allele (%)	homo	het	total (%)^a^	homo	het	total (%)^a^	homo	het	total (%)^a^	homo	het	total (%)^a^	
Jiangsu	102	26	10	36 (35.3)	12	18	42 (67.7)	2	7	11 (17.7)	1	7	9 (14.5)	0	0	0	30.4
Nei Mongol	115	30	5	35 (30.4)	14	18	46 (70.8)	0	11	11 (16.9)	0	3	3 (4.6)	1	3	5 (7.7)	28.3
Beijing	155	37	6	43 (27.7)	24	13	61 (76.3)	0	10	10 (12.5)	0	0	0	0	9	9 (11.3)	25.8
Hebei	64	14	3	17 (26.6)	7	9	23 (74.2)	0	3	3 (9.7)	0	1	1 (3.2)	0	4	4 (12.9)	24.2
Shanghai	31	7	1	8 (25.8)	3	5	11 (73.3)	0	2	2 (13.3)	0	1	1 (6.7)	0	1	1 (6.7)	24.2
Heilongjiang	36	5	4	9 (25.0)	1	7	9 (64.3)	0	2	2 (14.3)	0	0	0	0	3	3 (21.4)	19.4
Guangdong	77	15	4	19 (24.7)	10	7	27 (79.4)	0	4	4 (11.8)	0	0	0	0	3	3 (8.8)	22.1
Sichuan	109	17	8	25 (22.9)	10	13	33 (78.6)	0	3	3 (7.1)	0	4	4 (9.5)	0	2	2 (4.8)	19.3
Shanxi	57	11	2	13 (22.8)	4	9	17 (70.8)	0	5	5 (20.8)	0	1	1 (4.2)	0	1	1 (4.2)	21.1
Gansu	42	7	2	9 (21.4)	3	5	11 (68.8)	0	3	3 (18.8)	0	0	0	0	2	2 (12.5)	19
Jilin	57	12	0	12 (21.1)	7	4	18 (75.0)	0	5	5 (21.0)	0	0	0	0	1	1 (4.0)	21.1
Fujian	48	6	4	10 (20.8)	5	4	14 (87.5)	0	1	1 (6.3)	0	0	0	0	1	1 (6.3)	16.7
Ningxia	145	20	9	29 (20.0)	8	14	30 (61.2)	1	3	5 (10.2)	0	4	4 (8.2)	0	10	10 (20.4)	16.9
Xinjiang	136	19	8	27 (19.9)	9	5	23 (50.0)	2	4	8 (17.4)	0	3	3 (6.5)	1	10	12 (26.1)	16.9
Hubei	47	7	2	9 (19.1)	6	2	14 (87.5)	0	0	0	0	0	0	0	2	2 (12.5)	17
Yunnan	230	23	19	42 (18.3)	11	14	36 (55.4)	1	3	5 (7.7)	1	2	4 (6.2)	1	18	20 (30.8)	14.1
Guiyang	138	23	2	25 (18.1)	16	9	41 (85.4)	0	6	6 (12.5)	0	0	0	0	1	1 (2.1)	17.4
Henan	126	16	5	21 (16.7)	10	8	28 (75.7)	0	5	5 (13.5)	0	0	0	0	4	4 (10.8)	14.7
Tibet	118	0	19	19 (16.1)	0	2	2 (10.5)	0	1	1 (5.3)	0	0	0	0	16	16 (84.2)	8.1
Qinghai	56	5	3	8 (14.3)	1	3	5 (38.5)	2	2	6 (46.2)	0	0	0	0	2	2 (15.4)	11.6
Anhui	35	3	2	5 (14.3)	1	4	6 (75.0)	0	1	1 (12.5)	0	1	1 (12.5)	0	0	0	11.4
Shaanxi	52	3	2	5 (9.6)	1	4	6 (75.0)	0	1	1 (12.5)	0	0	0	0	1	1 (12.5)	7.7
Guangxi	87	1	5	6 (6.9)	1	4	6 (85.7)	0	0	0	0	0	0	0	1	1 (14.3)	4
total	2063	307	125	432	164	181	345	8	82	90	2	27	29	3	95	98	17.9

homo: homozygous; het: hetrozygous; ^a^percentage of total mutant alleles identified.

### Frame-shift and nonsense Pathogenic Mutations

The c.235delC is the most prevalent mutation in the Chinese NSHI population with a total of 509 alleles (164 homozygous, 113 compound heterozygous with other pathogenic mutant alleles, and 68 one heterozygous allele only), followed by 98 c.299_c.300delAT mutant alleles (8 homozygotes, 76 compound heterozygotes, and 6 one allele heterozygotes), 31 c.176_c.191del16 mutant alleles (2 homozygous, 25 compound heterozygous and 2 with only one allele), and 12 c.35delG mutant alleles(2 homozygous, 3 compound heterozygous and 2 with only one allele) (Supplemental Table 1). The four prevalent mutations account for 88.0% (650/739) of all mutant alleles identified. Five novel mutations were identified in 20 patients; including two nonsense; p.W3X, p.Y152X, and 4 frame-shift truncation mutations; c.99delT, c.155–c.158 delTCTG, and c.512–c.513 insAACG. Among these, c.512–c.513insAACG occurs in 12 patients, including one homozygous from Yunnan. The novel truncation mutations account for only about 3.1% (23/739, Supplemental Table 1) of the overall *GJB2 *mutant alleles. The most prevalent Caucasian mutation, c.35delG, was found in 2 homozygous, 3 compound heterozygous, and 5 single allele heterozygous patients. Among the patients with c.35delG, 70% of patients (7/10) are Uigur from Xinjiang area. The c.35insG mutation was found in 2 patients (both are Hui people) compound heterozygous with the c.235delC mutation. Other reported frame-shift mutations; 1 c.388–c.397del10 and 3 c.605–c.606ins46, as well as nonsense mutations; 3 p.E47X, account for a small fraction (1.0%) of *GJB2 *mutant alleles. Overall, 92.6% (684/739) of the pathogenic mutations are frame-shift truncation or nonsense mutations, and they are predicted to cause loss of function of connexin 26. Only 6.9% (51/739) of the mutant alleles are reported missense mutations.

### Reported missense pathogenic mutations

**Figure 2:**
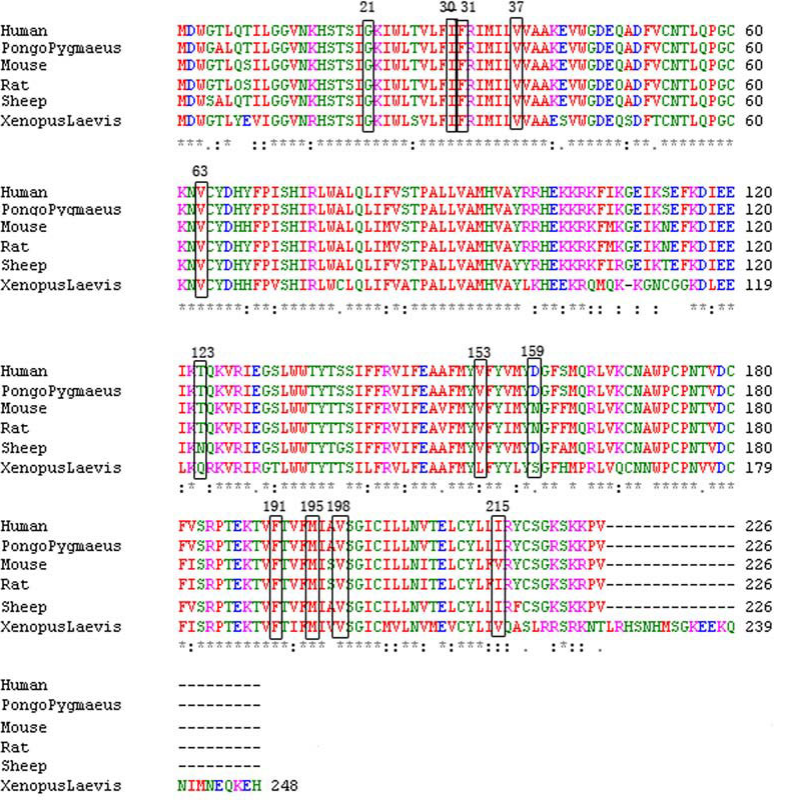
**Amino acid alignment of Connexin26 in different species**.

There are 8 reported missense pathogenic mutations and 1 in-frame deletion of 1 single amino acid, c.424_c.426del3 (p.del142F), which occurs in 4 heterozygous patients (Supplemental Table 1). The 8 missense mutations are p.G4D (3 heterozygous patients), p.R32C (one patient in compound heterozygosity with c.235delC), p.R143W (4 compound heterozygotes), p.T86R (all compound heterozygous, 6 with c.235delC and 1 with c.299_c.300delAT), p.R127H (one compound with p.Y152X, 31 single heterozygotes), p.S139N (2 single heterozygotes), p.E47K (one compound with c.299_c.300delAT), p.S85P (single heterozygote). All occur in an evolutionarily highly conserved region (Figure 2) [26,29,30].

### Unclassified Variants

Twelve unclassified missense variants were identified. The p.G21R is most likely to be pathogenic based on its highly evolutionarily conserved nature and the dramatic effect of the amino acid substitutions on structure and ionic strength. The p.I215N variant is located in the conserved region of C-terminal ion channel domain. Replacing the hydrophobic amino acid isoleucine with a hydrophilic amino acid asparagine in this conserved region is expected to cause detrimental effect. This variant is also in compound heterozygous with a novel pathogenic mutation, c.155_c.158delTCTG. Thus, it is likely to be pathogenic.

The missense variants, p.I30F, p.F31L, p.V63L, p.V153A, p.D159N, p.F191L, p.M195V, and p.V198M, do not involve drastic change in amino acid structure and polarity. They are all present as single heterozygous alleles without the presence of a second pathogenic mutant allele. Thus, their pathogenicity cannot be determined. Other changes of the same amino acids have been reported. For example, p.V63A has been reported as a novel variant, p.V153I and p.D159N were reported as a polymorphism [29]. The p.M195V and p.V198M, each occurs in two patients, without the second mutant allele. Each of the other variants occurs as heterozygous in one patient. None of these missense variants were detected in the control population.

### Uncharacterized Novel Silent Variants

Several nucleotide substitutions do not result in amino acid change. These are p.A49A, p. K61K, p.F146F, and p.T186T (p.T186T is heterozygous with a single c.235delC). Although these nucleotide changes do not alter the encoded amino acids, we cannot exclude the possibility that they may activate an exonic splice enhancer and cause aberrant splicing. Alternatively, changes in triplet codon may affect the preference of codon usage or the stability of the mRNA, which in turn can affect the protein levels.

### Genotypes and Carrier Frequency in the Normal Control Population

*GJB2 *is a small gene but harbors many mutations. Thus, the carrier frequency of *GJB2 *mutation in the Chinese population is not negligible. We sequenced the coding region of 301 normal control individuals of the Han ethnic group. Nine individuals were found to be heterozygous carriers of *GJB2 *pathogenic mutations; three had the c.235delC, three had the c.299_c.300delAT, and the c.512_c.513insAACG, c.35delG, and p.E47X mutation have been detected in single individuals (see Supplemental Table 3). Thus, the carrier frequency of *GJB2 *mutations in the control population is 3%.

### Frequencies of missense variants in patient and control populations

The frequencies of common missense variants such as p.V37I, p.V27I, p.I203T, p.T123N, p.E114G in patients, control, and other Asian populations were compared (see Supplemental Table 4 and Table 5). The pathogenic role of p.V37I has been controversial [24-26,30-33]. It was found that the p.V37I allele frequency was significantly higher in the Han patient group (excluding all cases with two clearly pathogenic mutations) than in the control group (6.7% and 2.8% respectively,. p = 0.0003), supporting a pathogenic role of p.V37I. The allele frequencies of p.V27I, p.E114G, p.I203T, and p.T123N were higher in the control group than in the Han patient group (excluding all cases with two clearly pathogenic mutations), arguing against their pathogenic role (see Supplemental Table 4 and Table 5).

### *GJB2 *mutation spectra among different sub-ethnic groups in China

As indicated in Table 2, the frequency of *GJB2 *mutations varies from 4% in Guangxi to 30.4% in Jiangsu. These results suggest that the variation in mutation frequencies may be due to ethnic diversity in various regions. The total population of China is 1.3 billion and sub-populations of Han, Tibetan, Hui, Man, Mon, minorities in Xinjiang, and minorities in South-western China are 1137.4 million, 5.4 million, 9.8 million, 10.7 million, 5.8 million, 10.8 million, and 57.1 million, respectively (, ). We therefore analyzed the mutation frequencies in different sub-ethnic groups. As shown in Supplemental Table 6, Hui has the highest frequency of overall *GJB2 *mutations, followed by Han and minorities in Xinjiang with 20.3, 19.1, and 15.3% respectively. Tibetan and the minorities in the Southwest have lower mutation frequencies, 9.4 and 5.0% respectively, similar to the frequencies observed in corresponding regions. The majority of mutations found in this study were found in the Han patient group (1640 cases) only except c.35 insG that was in compound heterozygous with c.235delC found in two Hui patients. The common Caucasian mutation, c.35delG was mainly detected in the minorities of Xinjiang, and accounted for almost half of the *GJB2 *mutant alleles in minorities of Xinjiang (9 c.35delG/19 total mutant alleles). The finding of the c.35delG mutation in Xinjiang may be due in part to the close vicinity of Xinjiang to Russia and Eastern European countries, and possible admixture. The Maan sub-ethnic group also appears to have diverse *GJB2 *mutation spectrum because mutations other than c.235delC account for more than one third of the mutant alleles. The three most common mutations c.235delC, c.299_c.300delAT, and c.176_c.191del16 account for 100% of *GJB2 *mutations in 18 Mongolian individuals analyzed. However, the sample size is too small to be statistically significant.

## Discussion

Previous reports have suggested that the prevalence of *GJB2 *mutations among different ethnic groups varies. In our patients, the most common Caucasian mutation, c.35delG was only found in 10 patients (seven of them were Uigur from Xinjiang). Instead, the c.235delC account for 68.9% of all *GJB2 *mutant alleles in our Chinese study population. These results support that the c.235delC mutation in connexin 26 gene is the most prevalent mutation in most Asian populations, including Han Chinese [11,24,30,34]. The results from this study indicate that analysis of four common mutations, c.235delC, c.299_c.300delAT, c.176_c.191del16, and 35delG can detect 88.0% (650/739) of *GJB2 *mutations. In 13 regions of China, by analyzing these four mutations, we were able to identified at least one mutant allele in all studied patients with one or two *GJB2 *mutations (see Table 2 and Supplemental Table 2). In contrast, mutations in the *GJB2 *gene account for a variable proportion of the molecular etiology of NSHI in different regions and sub-ethnic groups in China. Our results have tremendous impact on the design of molecular diagnostic and carrier testing of NSHI families in China. For example, in addition to the three most common mutations of c.235delC, c.299_c.300delAT, c.176_c.191del16, for minorities in Xinjiang, testing of Caucasian c.35delG mutation should be included. In patients with Maan ethnic background, sequencing of the *GJB2 *coding region should be offered, since the analysis of three common mutations detects only 71% of *GJB2 *mutant alleles. In minorities from Southwest provinces, although the three most common mutations account for >90% of all *GJB2 *mutations, defects in *GJB2 *gene account for only a small fraction (5%, Supplemental Table 2 and Table 6) of mutant alleles in NSHI patients. Thus, in these groups, analysis of other NSHI related genes should be pursued.

We recently reported that 7.8% of patients with autosomal recessive nonsyndromic hearing impairment in China were homozygous for the most common c.235delC mutation in *GJB2 *gene and 8.5% of them carried one mutant allele of the c.235delC mutation [28]. Sequencing of the coding region of the *GJB2 *gene reveals that 14.9% of the patients carry two pathogenic *GJB2 *mutation and 6.1% carry only one mutant allele. These results are comparable to other reported studies [7,11,13,24,29,30,33-35]. The proportions of patients with *GJB2 *mutations carrying only one mutant allele vary among different regions, different sub-ethnic groups, and different countries [7,11,13,24,29,30,33-35]. The observation that sequence analysis of *GJB2 *gene in subjects with autosomal recessive NSHI results in a high number of patients with only one *GJB2 *mutant allele has been puzzling [23]. Our unpublished data showed that no mutation were found in *GJB2 *Exon1 and its splicing sequence among 851 deaf individuals from Central China in this cohort which suggested extremely low detection rate of *GJB2 *Exon1 mutation among Chinese deaf population. For there is higher frequency of single heterozygous *GJB2 *mutation detected in the deaf population than in the normal population in this study, the further more extensive study of sequence change in *GJB2 *Exon1 or promoter area and 3'-UTR, fragment deletion neighboring *GJB2 *ORF region and digenic inheritance with other genes are already considered in this large Chinese deaf cohort for elucidating complex pathogenesis of *GJB2 *gene to hearing impairment. We already added a paragraph in discussion. Thus, a digenic hypothesis was proposed and mutations in two other connexin (Cx) genes, *GJB6 *for Cx30 and *GJB3 *for Cx31 were studied [21,22,36]. In families with clear evidence of linkage to the DFNB1 locus, which contains two genes, *GJB2 *and *GJB6 *[6,20], a common 309 kb deletion, involving the coding region *GJB6 *gene upstream of *GJB2 *gene has been identified and found to account for up to 10% of DFNB1 alleles in Caucasians [22]. We analyzed the deletion in *GJB6 *gene in 372 patients from Inner Mongolia and central China, and deletions in *GJB6 *gene were not detected. Similar studies of *GJB6 *mutations in Taiwanese prelingual NSHI patients carrying one *GJB2 *mutant allele also did not detect any deleterious mutations in *GJB6*, consistent with our results [30].

Although the spectrum of rare *GJB2 *mutations varies among sub-ethnic groups and in different regions of China, the same most common c.235delC mutation is shared. This observation is in agreement with the reports from the studies of other Asian NSHI patients [10,11,24,30,34]. However, instead of c.299_c.300delAT being the second most prevalent mutation, p.G45E accounts for 16% of the Japanese *GJB2 *mutations, while p.G4D accounts for 10.6% of Taiwanese *GJB2 *mutant alleles [10,30]. The p.G45E mutation was not detected in our patients. The p.G4D mutation accounts for only 0.3% of *GJB2 *mutant alleles in Chinese NSHI patients and was recently reported in a US study [29,30].

Among the 23 pathogenic mutations, 14 cause truncated connexin 26 proteins due to nonsense or frame-shift mutations, 8 are missense mutations, and one is a deletion of one amino acid. These mutations occur along the coding region. The truncation mutations account for 92.6% of the mutant alleles. Amino acids sequence homology alignment reveals that all missense mutations and unclassified variants occur at an evolutionarily conserved amino acid (Figure 2).

Three missense variants, p.V63L, p.V153A, and p.V198M, are located in extracelluar domain 1, 2, and transmembrane span 4, respectively, of connexin 26 protein. All these changes have not been reported in the Connexins and Deafness mutations database at . However, p.V63L has been found in 1 Taiwanese patient [30]. These three variants likely contribute to the pathogenesis of deafness, because (a) they were detected only in the patient group and not in 394 Japanese, 864 Taiwanese, 494 Korean and 301 Chinese (in this study) hearing normal subjects, and (b) they are evolutionarily conserved in xenopus, mouse, rat, sheep, orangutan, and human (Figure. 2). These variants were found in a heterozygous state in 4 unrelated patients who carried only one mutant allele.

The pathogenicity of p.V37I is controversial. In a recent multicenter study, the p.V37I mutation was found to be associated with mild to moderate hearing impairment (median 25–40 dB) [37]. Our study revealed that p.V37I with an allele frequency of 6.7% (185/2744) in the Han patient group (excluding all cases with two clearly pathogenic mutations) is significantly higher compared with that (2.8%;17/602) found in the control population (p = 0.0003, see Supplemental Table 4 and Table 5), supporting Wu's opinion to reassignment of p. V37I from an allele variant to a pathogenic mutation [38].

The p.T123N is an unclassified variant. It was counted as a mutation in Japanese group but a polymorphism in a Taiwanese study [10,30]. We found a higher p.T123N allele frequency in the control group than in the patient group, suggesting that it may be neutral variant. However, its clinical implication is not clear at this time.

The results of this study provide a great potential benefit for the clinical application of genetic testing for deafness. Based upon our preliminary data of molecular epidemiology of hearing impairment in China [28,39-41], Li has combined allele-specific PCR and universal array (ASPUA) methodologies for the detection of mutations causing hereditary hearing loss. It was employed for multiplex detection of 11 mutations in *GJB2*, *GJB3*, *SLC26A4 *and mitochondrial DNA causing hereditary hearing loss [42]. Although this simple screening chip only include probes and primers for the c.35delG, c.176_c.191del16, c.235delC, c.299_c.300delAT mutations of *GJB2 *gene, it can detect 88.0% (650/739) of *GJB2 *mutations among these 2063 deaf individuals, meanwhile, up to 88.9% (384/432) of 432 patients confirmed to carry at least one *GJB2 *mutation by sequencing in this study will be picked up by this fast screen method. The new methods for multiple mutation detection including ASPUA with capacity to test more gene loci have been under developed in our center, the data of this study will be crucial for the mutation selection in any new technology development for *GJB2 *gene testing in Chinese population.

In summary, this study revealed a unique *GJB2 *mutation spectrum in Chinese patients with nonsyndromic hearing impairment. The c.235delC mutation is the most frequent mutation in Chinese patients. Testing of four common mutations, c.235delC, c.299_c.300delAT, c.176_c.191del16, and c.35delG can detect 88.0% of the *GJB2 *mutant alleles. However, in some regions or sub-ethnic groups, the *GJB2 *mutations only account for a small fraction of the NSHI mutant alleles. In these regions, analysis of NSHI related genes is necessary. The molecular defects of more than 80% of the mutant alleles for NSHI in China remain to be identified.

## Competing interests

The authors declare that they have no competing interests.

## Authors' contributions

PD, FY and BH carried out the molecular genetic studies, participated in the sequence alignment and drafted the manuscript. GW, QL, YY, XL, KY, JH, JH, YH, YW, QY, YY, HL, LL, WD, XZ, YY, JC, NH, XX, JZ, LT, RS, YL, SS, RZ, HW and YM carried out epidemiological survey.

DK and XZ participated in the sequence alignment. SZHY and DH participated in the design of the study and performed the statistical analysis. PD, DH, XL and BW conceived of the study, and participated in its design and coordination and helped to draft the manuscript. L-JW reviewed and interpreted the results, drafted and revised the manuscript.
